# Four in one—Combination therapy using live *Lactococcus lactis* expressing three therapeutic proteins for the treatment of chronic non-healing wounds

**DOI:** 10.1371/journal.pone.0264775

**Published:** 2022-02-28

**Authors:** Jere Kurkipuro, Igor Mierau, Thomas Wirth, Haritha Samaranayake, Wesley Smith, Hanna-Riikka Kärkkäinen, Mirka Tikkanen, Juha Yrjänheikki

**Affiliations:** Aurealis Therapeutics, Kuopio, Finland; Daegu Gyeongbuk Institute of Science & Technology, REPUBLIC OF KOREA

## Abstract

Diabetes mellitus is one of the major concerns for health care systems, affecting 382 million people worldwide. Among the different complications of diabetes, lower limbs chronic ulceration is a common, severe and costly cause of morbidity. Diabetic foot ulcers are a leading cause of hospitalization in diabetic patients and its rate exceed the ones of congestive heart failure, depression or renal disease. Diabetic non-healing ulcers account for more than 60% of all non-traumatic lower limb amputations and the five-year mortality after amputation is higher than 50%, being equal to several types of advanced cancer. The primary management goals for an existing diabetic foot ulcer are to achieve primary healing as expeditiously as possible and to achieve a reduction of the amputation rate in the patients. Unfortunately, approximately a quarter of patients do not partially or fully respond to the standard of care. Advanced therapies for chronic wounds are existing, however, recent guidelines including the latest reviews and meta-analyses of the scientific and clinical evidence available from current treatment strategies and new therapeutic agents revealed that there is a lack of clinical data and persistent gap of evidence for many of the advanced therapeutic approaches. In addition, no pharmacological wound healing product has gained authority approval for more than 10 years in both US and EU, constituting a highly unmet medical need. In this publication we present data from a live biopharmaceutical product AUP1602-C designed as a single pharmaceutical entity based on the non-pathogenic, food-grade lactic acid bacterium *Lactococcus lactis* subsp. *c*r*emoris* that has been genetically engineered to produce human fibroblast growth factor 2,interleukin4 and colony stimulating factor 1. Designed to address different aspects of wound healing (i.e. fibroblast proliferation, angiogenesis and immune cell activation) and currently in phase I clinical study, we show how the combination of the individual components on the wound micro-environment initiates and improves the wound healing in chronic wounds.

## Introduction

Diabetes mellitus (DM) is a major concern for health care systems. In 2019 463 million adults worldwide were affected, and the number of the people with DM is estimated to rise to 700 million by 2045 [1–3] Patients with DM have an increased risk of further complications such as nephropathy, retinopathy, cardiovascular diseases or peripheral neuropathy, which in turn can contribute to the development of diabetic foot ulcers (DFUs) [4, 5]. Chronic ulceration is the most frequently occurring complication of DM with a lifetime incidence of DFU of between 19 and 34% in DM patients [1, 6, 7]. On the basis of 2017 prevalence data from the International Diabetes Federation, Armstrong et al. estimated that foot ulcers are developed annually in slightly more than in 2% DM patients and between 5–7.5% in patients with neuropathy worldwide [1]. DFUs are a leading cause of hospitalization in DM patients; its rate exceeds that of congestive heart failure, depression and renal disease [8]. Non-healing diabetic ulcers account for more than 60% of all non-traumatic lower limb amputations [9]. Furthermore, the five-year mortality after diabetes-related amputation is 30.5%, closely equalling pooled 5-year survival of all reported cancer cases at 31% [10].

The physiological process of wound healing involves a complex interplay between many cellular players of the skin, primarily keratinocytes, fibroblasts, endothelial cells of vessels and recruited immune cells, and their associated extracellular matrix in a well-orchestrated cascade through hemostasis, inflammatory, proliferative and maturation phases [11, 12]. However, in chronic wounds such as DFU, the tightly controlled process of wound healing is impaired and detained in one or more phases. Several pathogenic abnormalities, ranging from disease-specific intrinsic flaws in blood supply, angiogenesis, and matrix turnover to extrinsic factors due to plasma cells and their related pro-inflammatory cytokines in diabetic wounds contribute to the onset of a pro-degradative micro-environment, which results from the imbalance between matrix synthesis and degradation. Especially chronic inflammation seems to play a major role in the pathogenesis of DFU [3, 13].

In acute wounds, the inflammatory phase is followed by the emergence of, or polarization of the macrophage population into the anti-inflammatory, pro-repair M2-type macrophages suppressing the inflammation as well as recruiting endothelial cells and fibroblasts enhancing the ECM formation, angiogenesis, re-epithelialization and wound closure, and effectively transitioning the wound healing from the inflammatory to the proliferative and remodelling phases [3]. In chronic wounds however, the shift of the M1-type macrophages to the alternative M2-type macrophages does not readily occur; the wound remains in the state of inflammation, which is inhibiting the initiation of the proliferation phase and subsequent tissue regeneration [3, 14].

The primary management goals for an existing DFU are to achieve healing as expeditiously as possible and to reduce amputation rates in this challenging patient group. The standard of care (SoC) in DFU management is a multi-disciplinary treatment regimen consisting of, for example, metabolic control of DM, cleaning and appropriate debridement of non-viable tissue on the ulcer, decrease of mechanical pressure with proper offloading, control of wound infections, ensuring adequate lower-extremity blood inflow, and application of diligent local moist wound care with appropriate management of wound exudation. In addition, attention is paid to correct treatable malnutrition, vitamin- and micronutrient deficiencies, to manage oedema and treating other comorbidities, if present. When treated appropriately, DFUs heal in many patients within the first few months, preventing the need for amputation [15]. Unfortunately, approximately one quarter of patients do not respond (partially or fully) to the SoC treatment for DFU [15], and the estimated rate of recurrence after ulcer healing is about 40% within one year, almost 60% within 3 years and 65% within 5 years [1]. Predictors of non-healing ulcers include advanced age, male sex, heart failure, the inability to stand or walk without help, end-stage renal disease, larger ulcer size, peripheral neuropathy and peripheral arterial disease [15].

Advanced therapies are a promising alternative and one of the fastest growing markets for treatment of chronic wounds and includes devices or products such as negative wound pressure therapy [16, 17], hyperbaric oxygen therapy [18], extracorporeal shock wave stimulation [19], special purpose dressings [20–22], skin grafts and bioengineered skin [23], drugs or biologics such as locally administered growth factors [24]. Regarding pharmacological and/or medicinal wound healing products for DFUs, so far only growth factors and tissue-based substitution components have gained approval in the US and other major countries. These include the recombinant human platelet-derived growth factor Regranex^®^ (Ortho-McNeil-Janssen Pharmaceuticals) [25]; three recombinant human epidermal growth factors (rhEGF): Easyef^®^ (Daewong Pharmaceuticals) [26], Heberprot-P^®^ (Heber Biotec) [27], and Regen-D™150 (Bharat Biotech) [28]; the bilayered living skin construct Apligraft^®^ (Organogenesis Inc) [29]; the human fibroblast-derived dermal substitute Dermagraft^®^ (Organogenesis Inc) [30]; and the bilayer dermal regeneration matrix Omnigraft^®^ (Integra Lifesciences) [31]. Among them, only Regranex^®^ (becaplermin) had achieved EU approval in 1999 but was voluntarily withdrawn in 2012 by the applicant for commercial reasons. In the US, this product is still approved.

We have developed a novel four-in-one recombinant live biopharmaceutical product (LBP), named AUP1602-C, to induce tissue regeneration in chronic inflammatory wounds such as DFU. The product is currently ongoing a clinical Phase I study to evaluate safety and efficacy in patients with chronic non-healing DFU. The drug product AUP1602-C is based on a genetically modified Gram-positive bacterium *Lactococcus lactis* subsp. *cremoris* expressing three human therapeutic proteins, namely fibroblast growth factor 2 (FGF-2), interleukin-4 (IL-4) and colony-stimulating factor 1 (CSF-1). The aim of this approach is to modulate the local micro-environment in the wound and to activate the distorted immune system present in DFU in order to initiate wound healing. More specifically, AUP1602-C is designed to 1) activate the immune system, 2) induce angiogenesis and 3) induce tissue regeneration (fibroblast proliferation).

The synergistic effect of the three therapeutic proteins produced by AUP1602-C together with the intrinsic immune-modulating activity of the bacteria on the wound micro-environment directly aims at initiating and promoting wound healing and at accelerating wound closure in chronic wounds.

In this publication, we present the preclinical data regarding the use of AUP1602-C in the well-established db/db diabetic mouse model of delayed wound healing indicating significant improvements in the key parameters of wound healing. Furthermore, we evaluate the minimal effective dosing regimen as well as assess the feasibility and safety of AUP1602-C treatment in GLP safety and toxicity studies. In essence, this publication shows for the first time the proof-of-concept of the treatment of chronic wounds by multi-therapy administered via single LBP successfully producing three therapeutic proteins for the treatment of chronic wounds in live animals.

## Materials and methods

### Strain

The bacterial strain used for the construction of drug product AUP1602-C was *Lactococcus cremoris* AUC1000, an *alr* deletion derivative of *L*. *cremoris* MG1363 [32, 33].

### Plasmid

**Fig 1:**
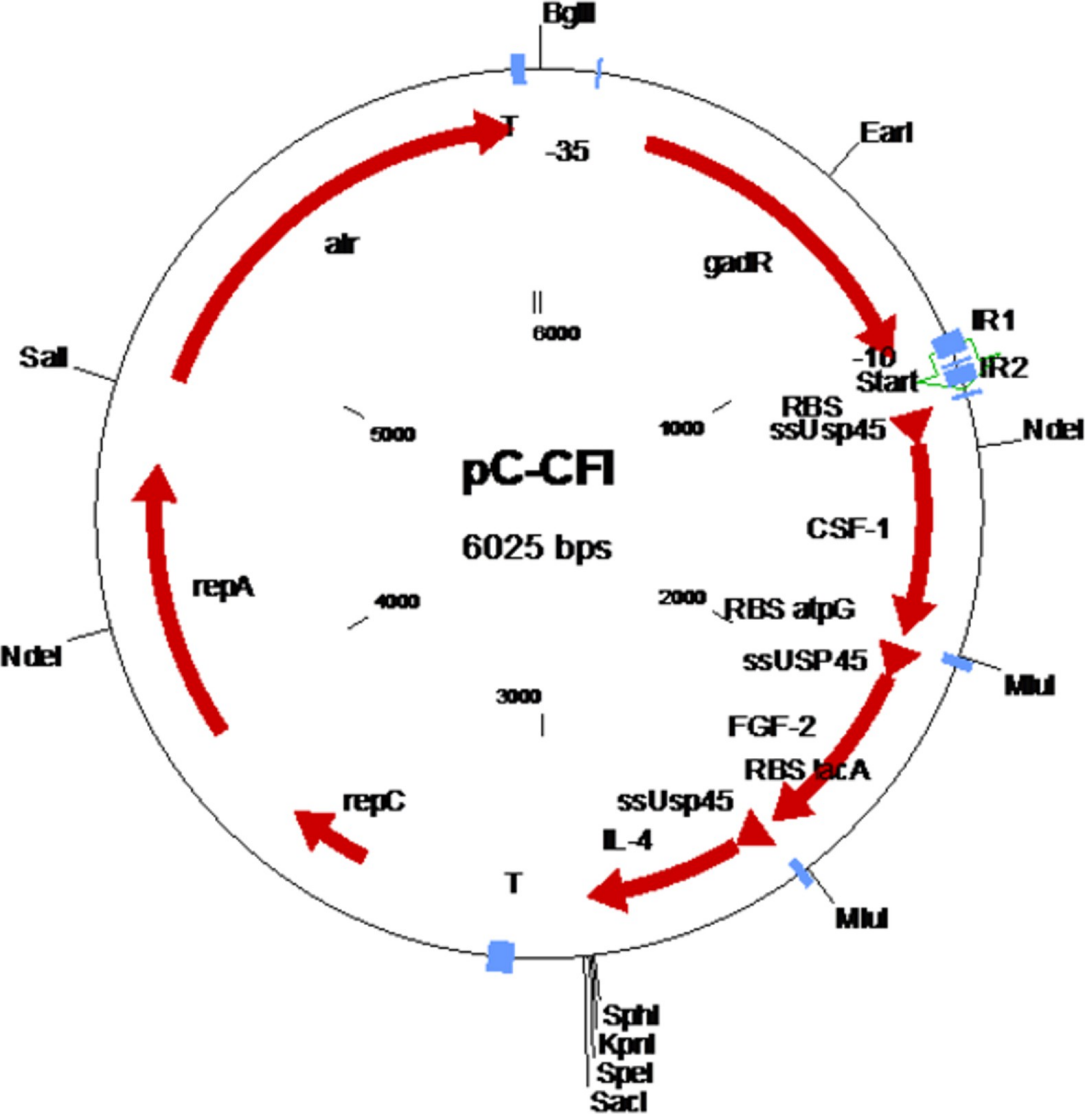
Schematic representation of plasmid pC-CFI. repA and repC: plasmid replication; alr: alanine racemase food grade selection gene; T: terminator; gadR: *gadR* regulator gene of the *Lactococcus cremoris gad* operon; ssUsp45: signal sequence of *L*. *cremoris usp45* gene; CSF-1: gene for human colony stimulating factor 1; FGF-2: gene for human fibroblast growth factor; IL-4: gene for human interleukin 4; RBS: ribosome binding site.

Plasmid pC-CFI (Fig 1) contains an *alr* gene for plasmid selection [33], the *gadCB* operon promoter cassette including the *gadR* regulator gene [34, 35] and the three human therapeutic genes chosen for this study optimized for expression in *L*. *cremoris* and arranged in an operon structure. The three human therapeutic genes are FGF-2, IL-4 and CSF-1.

The nucleotide sequence for human FGF-2 was derived from the amino acid sequence of FGF-2 isoform-3 (Uniprot P09038-2). Using SignalP 4.1 Server-analysis for Gram-positive bacteria [36] the N-terminal methionine and alanine residues were removed to improve the efficiency of cleavage of the signal peptide. The removal of these two amino acids was considered acceptable because the nine N-terminal amino acids of FGF-2 are cleaved *in vivo* by extracellular proteolytic degradation to produce biologically active FGF-2 [37].

The nucleotide sequence for human IL-4 was derived from the amino acid sequence of IL-4 isoform-1 (Uniprot P05112-1) using the 129-amino acid sequence of the active protein (amino acids 25–159). Using Signal P 4.1 Server-analysis for Gram-positive bacteria [36] an alanine residue was added to the N-terminus to improve the efficiency of cleavage of the signal peptide.

The nucleotide sequence for human CSF-1 was derived from the amino acid sequence of CSF-1 (Uniprot P09603-3) using the amino acids 33–190 [38]. Using Signal P 4.1 Server-analysis for Gram-positive bacteria [36] an alanine residue was added to the N-terminus to improve the efficiency of cleavage of the signal peptide.

The resulting amino acid sequences were converted to nucleotide sequences using the codon usage of *Lactococcus lactis*.

For the simultaneous expression of the three therapeutic human proteins an operon was designed containing the optimized genes for CSF-1, FGF-2 and IL-4 under the control of the P*gadC* promoter cassette. In order to facilitate the secretion of the therapeutic proteins each gene was coupled to the *L*. *cremoris* Usp45 signal sequence [39]. Finally, each gene was preceded by a ribosome binding site derived from *gadC* (first gene) [35] and the *atpG* [40] and *lacA* [41] genes of *L*. *cremoris*. Cloning was carried out using standard methods and *de novo* synthesized P*gadC* promoter cassette and the designed operon (Base Clear, Leiden, the Netherlands) [42]. The final construct, plasmid pC-CFI was transformed [43] into host strain AUC1000 generating product AUP1602-C [44].

### Drug product production

A master cell bank was produced (Biomay AG, Vienna, Austria) after single colony isolation of initially isolated colonies of the cloning process. One vial of the master cell bank was used to inoculate pre-culture 1, which was used to inoculate pre-culture 2. Subsequently, pre-culture 2 was used to inoculate the main 200-L fermentation in a single-use fermenter set-up. The culture was grown to stationary phase at 30°C and with pH regulation at pH 6.5. After harvest the culture was diafiltrated against a 2.5% Na-glutamate pH 6.5 buffer and concentrated to a final concentration factor of 20 times. Finally, the concentrated cell suspension was mixed 1:1 with the formulation- and cryoprotectant buffer of 2.5% Na-glutamate, 15% glycerol (end concentration). This suspension was filled at 2 ml into 20 ml glass vials and frozen at -70°C (Wacker Biotech, Amsterdam, the Netherlands).

### Other materials

The reconstitution solution (batch ID APL-0917-5991-03), was produced by Aesica Pharmaceuticals, UK, for the reconstitution of the AUP1602-C and was also used in the studies as vehicle. The reconstitution solution is a benign hypertonic solution containing 5% dextrose, 2.5% saline and 1.6% sodium acetate (pH 6.0–6.5).

Antibodies used in the western blotting and immunohistochemical stainings were rabbit anti-FGF2 (ab246354, 1:70, Abcam, UK), rabbit anti-IL-4 (ab9622, 1:70, Abcam, UK), rabbit anti-CSF-1 (ab9693, 1:80, Abcam, UK), rat anti-neutrophil NIMP-14 (ab2577, 1:100, Abcam, UK), rat anti-macrophage F4/80 (ab16911, 1:200, Abcam, UK), rabbit anti-CD31 (ab28364, 1:50, Abcam, UK), rat anti-BrdU (ab6326, 1:500, Abcam, UK).

### Western blots

In the western blots performed in Covance (Huntingdon, UK), the AUP1602-C stock vials were thawed and mixed with reconstitution solution in order to transfer 1x10^11^ CFU from each vial into growth media for 24 hours (+30 ± 2°C with gentle mixing). Following this, the secreted FGF-2, IL-4 and CSF-1 proteins in the culture growth medium were concentrated using TCA and acetone precipitation following which the proteins were electrophoretically separated on an appropriate polyacrylamide gel based on their size. Subsequently the proteins were electrotransferred onto an immobilizing membrane. Once unoccupied sites on the membranes were blocked, each membrane was incubated with the corresponding specific primary antibody. A secondary antibody coupled to horseradish peroxidase was then applied which upon addition of an appropriate substrate produces a complex that will luminesce and provide a signal that can be captured with the imaging system.

### *In vivo* studies

All in vivo studies performed for the data presented in this publication, were specifically approved by local ethics committee or Institutional Care and Use Committees. Diabetic mice studies were performed in CICA Biomedical (York, UK) using the BKS.Cg-m Dock7^m^ +/+ Lepr^db^ /J mice (Stock Code 00642, Jax, USA) aged approximately 8–9 weeks. Animals were allowed to acclimate for one week prior to the start of the study. Animals were maintained by the specific requirements of diabetic animals according to Home Office regulations and studies were approved by the Animal Welfare and Ethical Review Committee of the University of Leeds (Leeds, UK). For the full thickness wound, all mice were anaesthetised using isofluorane and air; and their dorsal flank skin was clipped and cleansed. A single standardised full-thickness wound (10mm x 10mm) was created on the left flank. Each wound was then photographed with an identification plate and calibration rule. All wounds were then dressed with the transparent film dressing Tegaderm™ Film (3M Deutschland GmbH, Germany) and animals were then allowed to recover in a warmed environment (+34°C). Animals were later restrained and dosed with one of the treatments described below (Table 1) applied topically by injection through the Tegaderm™ film onto the wound bed surface using a 27-gauge needle. All wounds were closely monitored for excessive build-up of applied agents and excessive wound site exudation. Excess product/fluid was removed by aspiration, prior to re-application of treatments.

**Table 1:** Compiled study groups and dosing regimen in the *in vivo* efficacy, dose finding and GLP safety & toxicity studies.

Study	Animal model	Wound size	Group	*n*	CFU/ml	Volume	CFU/wound	Regimen	# of doses
In vivo efficacy	Full thickness excisional wound in db/db diabetic mice	10x10 mm	Formulation vehicle	*10*	0	50 μl	0	Daily	7
			AUP1602-C	*10*	5x10^8^		2.5x10^7^		
Dosing frequency	Full thickness excisional wound in db/db diabetic mice	10x10 mm	Formulation vehicle	*10*	0	50 μl	0	Daily	7
			AUP1602-C	*6*	5x10^6^		2.5x10^5^	Daily	
					5x10^7^		2.5x10^6^		
					5x10^8^		2.5x10^7^		
					5x10^9^		2.5x10^8^		
					5x10^6^		2.5x10^5^	Every other day	
					5x10^7^		2.5x10^6^		
					5x10^8^		2.5x10^7^		
					5x10^9^		2.5x10^8^		
					5x10^6^		2.5x10^5^	Every fourth day	6
					5x10^7^		2.5x10^6^		
					5x10^8^		2.5x10^7^		
					5x10^9^		2.5x10^8^		
GLP Safety and toxicity	Full thickness excisio0nal wound in Göttingen minipigs	20x20 mm	Control (main)	*6*	0	200 μl	0	Every other day	7
			AUP1602-C (main)		5x10^7^		1x10^7^		
					5x10^9^		1x10^9^		
			Control (recovery)	*4*	0		0		
			AUP1602-C (recovery)		5x10^7^		1x10^7^		
					5x10^9^		1x10^9^		

All animals were terminated following wound photography on the final day of the studies. Termination was achieved by a UK Home Office ‘Schedule 1’ compliant method. For relevant groups, animals received an i.p. injection (30μg/g) of 5-bromo-2′-deoxyuridine (BrdU, Sigma) two hours prior to termination in order to facilitate immunostaining of proliferating cells. Following the sacrifice, wounds (with surrounding normal tissue) were excised and fixed in 10% neutral buffered formalin (Sigma, UK). Once fixed, tissue was bisected cranio-caudally and processed to paraffin wax for sectioning.

### (Immuno)histochemistry and image analysis

Representative sections of all collected mice wound tissues were de-waxed, antigen recovered (where necessary) and either stained with haematoxylin/eosin or picro-sirius red stainings, or immunostained with antibodies listed above using standard staining procedures for paraffin-embedded sections. Stained sections were digitally scanned and six regions of interest (each 250μm x 250μm) spanning the width of the wound were extracted using Aperio ImageScope software (Leica Biosystems). The number of neutrophils and proliferating cells, and the pixel area occupied by macrophages, neo-vascular structures, and collagen within each region of interest was then measured using Image Pro Plus software (Media Cybernetics, USA). Average measures for each parameter were calculated for each wound and for each of three wound regions (outer, intermediate and central).

### Wound closure image analysis

Image Pro Plus image analysis software (version 4.1.0.0, Media Cybernetics, USA) was used to calculate wound closure from scaled wound images taken at each assessment point. As the process of wound closure results from the combined effects of wound contraction and re-epithelialisation, wound closure over time was also considered with respect to these components. Furthermore, all wounds were visually assessed on a daily basis to establish their “healing” status where each wound was scored as to whether or not it displayed neo-dermal tissue generation activity by two independent observers. Neo-dermal tissue formation was considered to have initiated when blood vessels within the fascia of the wound base were concealed by overlying material. In addition, as an indicator of angiogenic activity, the wound images were also visually assessed and scored in terms of the level of ‘redness’ at the base of the wound.

### Statistical analysis

Fisher’s exact test (for proportionate data) was used to analyse the impact of treatment on initiation of neo-dermal tissue repair activity. Non-parametric analysis (Kruskall Wallace multivariate analysis followed by ad hoc two sample Mann Whitney U-test analysis) was used to test the significance of any inter-group differences in wound closure including the individual components for contraction & re-epithelialisation, as well as the histological parameters to be investigated in this study.

### Safety and toxicity

The GLP safety and toxicity study was performed in Citoxilab (Lille Skensved, Denmark) under Executive Order No. 1245 on GLP for Medicinal Products of Goold Laboratory Practise and the conduct of experimental animals was approved by the Animal Ethics Council (Rådet for Dyreforsog) in ccordance with the Danish Law on animal Experimentation (LBK nr. 474 15/05/2014, BEK nr. 2028 14/12/2020 and European Directive 2010/63/EU). The study used a total of 30 Göttingen SPF minipigs (15 males and 15 females) from Ellegaard Göttingen Minipigs A/S, DK-4261 Dalmose, Denmark. The animals were 19.5–24.2 kg and approximately 9–12 months at arrival. A pre-treatment period of approximately 3 weeks (including an acclimatisation period of 5 days) was allowed during which the animals were observed daily in order to reject animals in poor condition. For the wounding, the animals were anaesthetised with 40 mg azaperone/mL (1 ml/20 kg) and 1 mg atropine/mL (0.05 mL/kg) given as a single intramuscular injection. This was followed by an intramuscular injection in the neck (1.0 ml/10 kg body weight) of a mixture of Zoletil 50®Vet., Virbac, France (125 mg tiletamine and 125 mg zolazepam), 20 mg xylazine/mL (6.25 ml), 100 mg ketamine/mL (1.25 ml) and 10 mg butorphanol/mL (2.5 mL). Analgesia was initiate prior surgery and continued 24 hours afterwards with intramuscular injection of 0.3 mg buprenorphine/ml (0.04 ml/kg). In addition, 20 mg meloxicam/ml (0.02 ml/kg) was administered once a day for two days starting on the day of wounding.

The dorso-lateral area of both sides of the back of the animal was shaved (using shaving foam and a razor), washed with soap and water, disinfected with 70% ethanol which was rinsed off with sterile saline and finally dried with sterile gauze. A total of four circular full-thickness wounds (diameter 20 mm) were made on the prepared area of each animal using a specially designed circular cutter. After wounding, each wound was dressed with film dressing (Mepore, Mölnlycke®, 6 x 7 cm) and followed by a secondary foam dressing (Allevyn,Smith & Nephew, 10 x 10 cm) which was fixed by Fixomul® (BSN Medical) and covered by a gauze dressing. The dressings and the gauze bandage were retained by a netlike body stocking (Elastofix, BSN medical GmbH, Hamburg, Germany) attached to a neck collar. The dosing was performed by injection through the Mepore film dressing, according to the treatment schedule (Table 1). Following this a second piece of Mepore film dressing was applied ontop to cover the hole from the injection.

Necropsy was performed on all main animals on day 14 and on recovery animals on day 28. On the day of necropsy, the animals were weighed, examined externally and anaesthetised by an intramuscular injection in the neck or in the left hind leg (about 0.3 mL per kg body weight) of a mixture of Zoletil 50 Vet., Virbac, France (125 mg tiletamine and 125 mg zolazepam), 20 mg xylazine/mL (6.25 mL), 100 mg ketamine/mL (1.25 mL) and 10 mg butorphanol/mL (2.5 mL). The animals were euthanized by exsanguination. In the recovery period, starting on Day 14, the wounds were dressed as described in section however a Mepore film dressing was not applied. As the wounds were re-reepithelialised within the first week of recovery, dressing changes were halted and no further dressings were applied thereafter.

During the safety and toxicity study a detailed macroscopic evaluation of each wound was performed (scored for inflammation of wound edges and skin surrounding the wounds, haemorrhage, exudation, presence of necrotic tissue, granulation and presence of hypergranulation, oedema and abscess formation) and photographs of the wounds were taken. In addition, the clinical condition, body weight, food consumption, dose formulation analysis, haematology, clinical chemistry, urinalysis, ophthalmoscopic examinations, ECG measurements, biodistribution of the bacteria in blood and tissue, shedding assessment, macroscopic examination at necropsy, organ weight investigations and histopathological examinations were undertaken. All the described methods above are considered as routine approaches with the exception of the qPCR method for detection of AUP1602-C in the tissue samples which was specifically developed and validated for purpose (other data and methods not shown).

### qPCR

For the analysis of the collected tissues samples during the safety and toxicity study, a nested TaqMan^®^ PCR (qPCR) assay was validated (Accelero Bioanalytics GmbH) for detection and quantification of AUP1602-C. Thus, the test method comprised the exponential PCR amplification of a specific DNA product by means of a specific forward and a reverse primer set in combination with a thermostable DNA polymerase. A labelled and specific probe was able to hybridize at a defined position on the amplicons between the two primers. The selective enrichment of the amplicons was monitored by the DNA polymerase mediated hydrolysis of the hybridized probes, which generated a specific fluorescence signal in real-time (5´-exonuclease assay). The lower limit of quantification for the validated assay was 40 CFU and the limit of detection was 10 CFU.

## Results and discussion

### Western blot analysis of AUP1602-C secreted therapeutic factors

**Fig 2:**
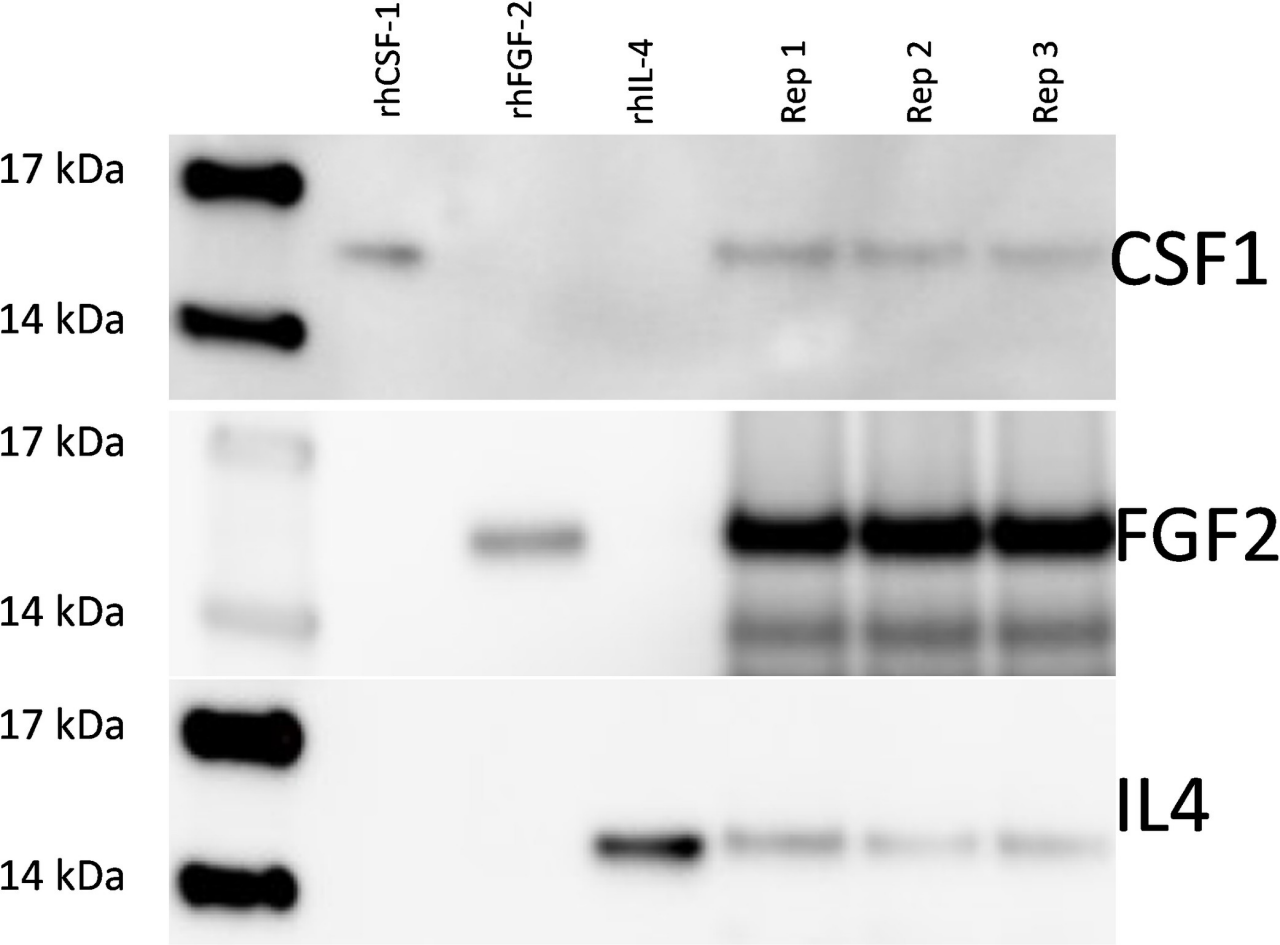
Western blots showing the three therapeutic proteins secreted by AUP1602-C. Lane 1: molecular weight marker; lanes 2–4: human recombinant protein positive controls; lanes 5–7 three replicate samples (Rep 1–3) of the AUP1602-C induced culture supernatants. Data not statistically analysed.

Prior to the *in vivo* experiments, the GMP grade AUP1602-C bacterial cryo-cultures were analysed with a series of release assays including Western blots. The Western blot assays were performed for AUP1602-C culture supernatant samples to demonstrate the secretion of the therapeutic factors. Fig 2 shows the presence of positive bands, which have the same size as the positive controls of the respective recombinant human proteins. These results show that all three proteins are produced, secreted and correctly processed by the signal peptide peptidase.

### Wound healing and efficacy in the diabetic db/db mouse model

**Fig 3:**
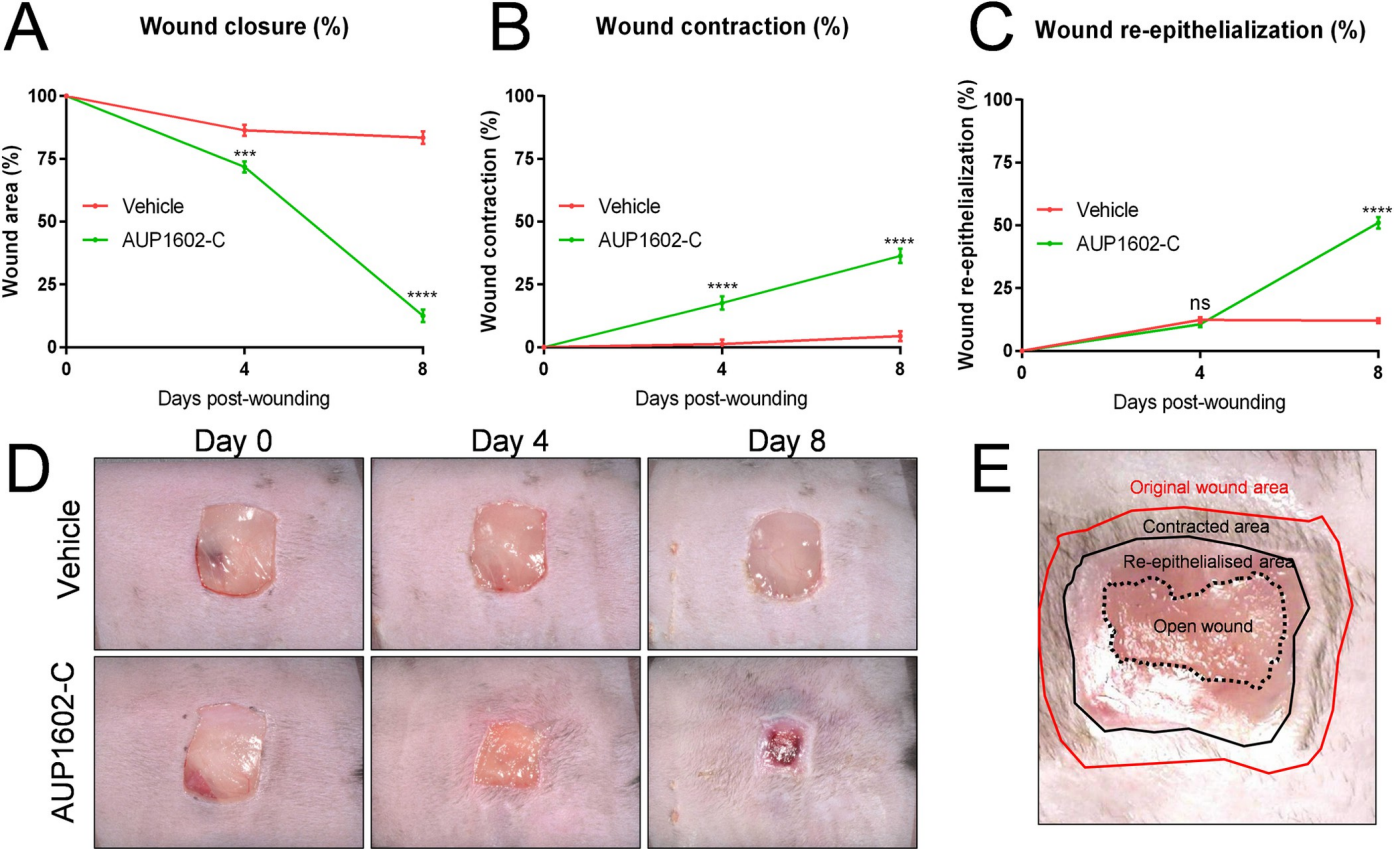
Measurements related to *in vivo* wound healing efficacy during treatment with AUP1602-C or vehicle. A) wound closure, B) wound contraction, C) wound re-epithelialization, D) representative images of the wounds during the treatment period and E) schematic indicating how wounds were analysed. All data is presented as mean ± SEM. Statistical analysis is performed with Kruskall Wallace multivariate analysis followed by ad hoc two sample Mann Whitney U-test analysis). ns = non-significant, *p < 0.05, **p < 0.01, ***p < 0.001 and ****p < 0.0001.

To assess the wound healing efficacy of the recombinant LBP AUP1602-C, the well-established db/db diabetic mouse model of delayed wound healing was used. After surgically excising the initial wound in the flanks of each mouse, the animals were then treated either with vehicle or AUP1602-C (5x10^8^ CFU/ml) applied topically onto the wounds daily for a duration of one week. Each wound was digitally photographed together with an identification/calibration plate immediately after injury and subsequently on days 4 and 8. For a given wound at a given time point the wound closure was expressed as the percentage of the remaining wound area relative to the initial wound area immediately after injury (*i*.*e*. day 0). Fig 3A (see also S1 Appendix for supportive developmental data) shows that the wounds in the mice that were treated with AUP1602-C were healing significantly more rapidly than the wounds of mice receiving the vehicle (p < 0.001 and p < 0.0001 on days 4 and 8, respectively).

**Fig 4:**
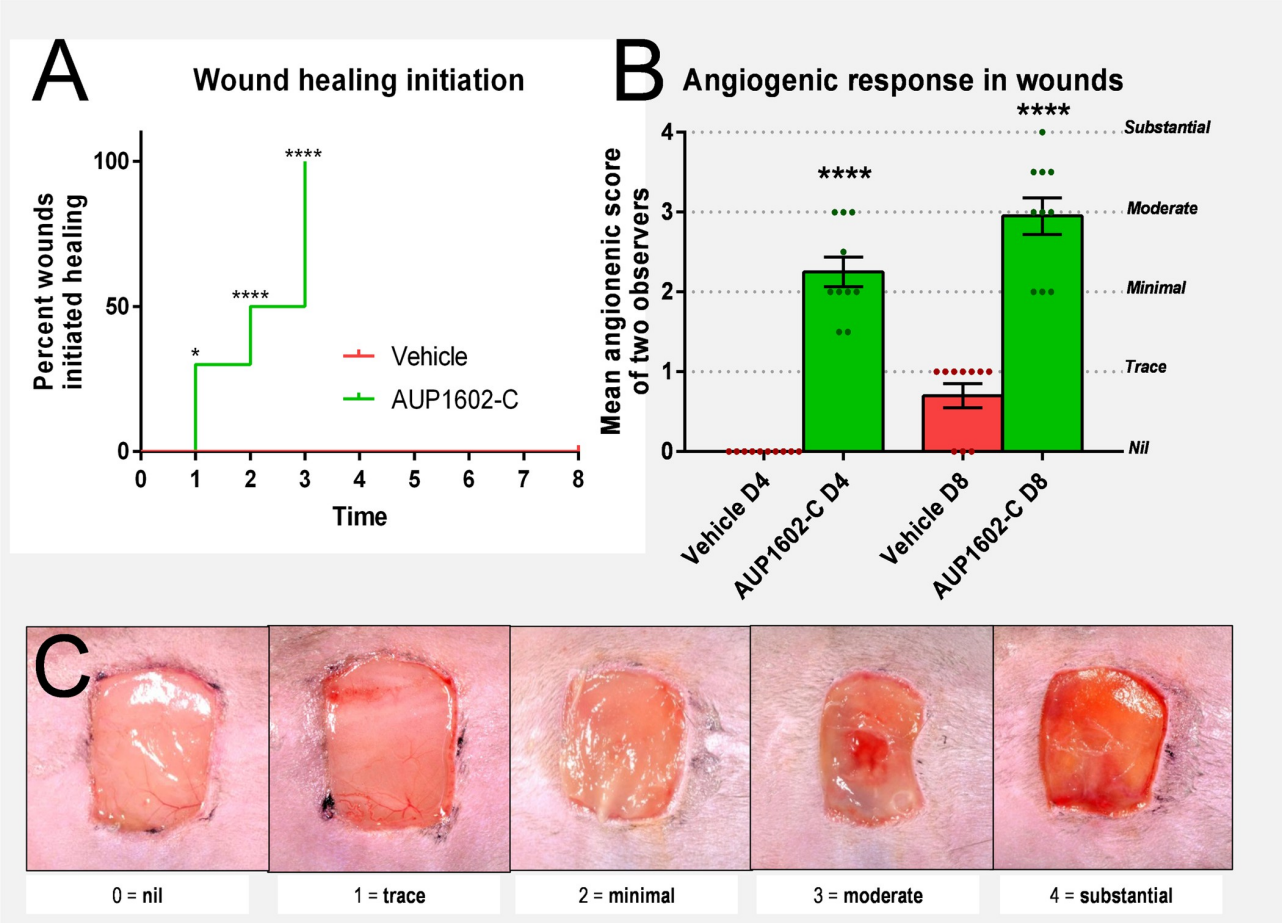
Initiation of wound healing and visual assessment of the angiogenic response during the treatment of the wounds with AUP1602-C or vehicle. A) wound healing initiation, B) scoring of visual assessment of angiogenic response with green and red dots indicating the individual mean score from two observers and C) example images for defining the clinical score regarding the visual assessment of angiogenesis. All data are presented as mean ± SEM. Statistical analysis was performed with Fisher’s exact test. ns = non-significant, *p < 0.05, **p < 0.01, ***p < 0.001 and ****p < 0.0001.

In addition to wound closure, the formation of the granulation tissue in the wound bed, which is driven by fibroblast cells that cause a centripetal compacting movement of the wound margins and thus contract the wound, was significantly (p < 0.0001 for day 4 and day 8) increased after treatment with AUP1602-C (Fig 3B). To complement the physical measurements of the wound closure and movement of margins, the wounds were visually assessed and scored by two independent observers to establish the wounds healing status. To this end the wounds also were scored for their dermal tissue generation activity (i.e. healing initiation) on a daily basis, their angiogenic response was scored on days 4 and 8 and the mean scores were compared between the study groups (Fig 4A and 4B). Increased dermal tissue generation activity was evident in 30% of the wounds receiving AUP1602-C on the day following the wounding, 50% by post-wound day 2 and visible in all wounds in the group by post-wound day 3. Control animals treated with vehicle showed no initiation of wound healing throughout the whole monitoring period of 8 days (Fig 4A). Furthermore, the angiogenic response scored significantly higher in animals treated with AUP1602-C since the majority of treated wounds displayed at least a general reddening of the wound base with indications of increased vascularization of the wound in comparison to the lack of effect or only traces of reddening in the wounds treated with the vehicle alone (Fig 4B).

### Histology and immunohistochemistry

To further investigate the treatment effect of AUP1602-C in the wound healing process on a microscopic level, a series of histological and immunohistochemical staining’s were performed on various wound samples. The staining’s were analysed in order to assess the different cellular properties involved in the wound healing process between animals that were treated with either vehicle or AUP1602-C. Tissue samples were analysed for granulation tissue depth (GTD), for the prevalence of neutrophils, macrophages, neo-vascular structures and proliferating cells as well as for the amount of collagen deposited within the tissues.

**Fig 5:**
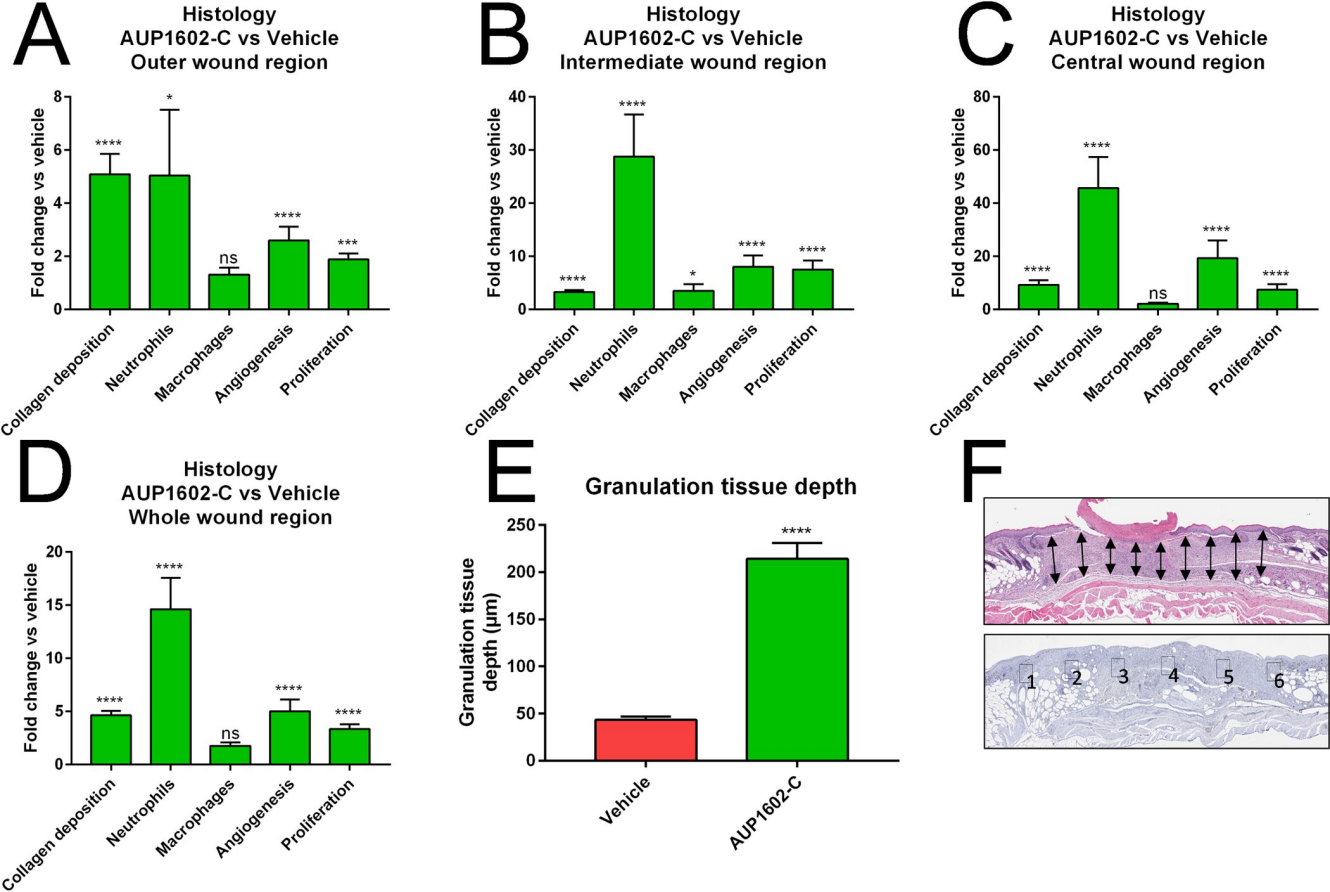
The protein expression fold changes in collagen deposition, neutrophile count, macrophage presence, angiogenesis and cellular proliferation. Fold changes measured from histological and immuno-histological staining’s after treatment with AUP1602-C in A) outer wound region, B) intermediate wound region, C) central wound region, D) whole wound area. In addition, E) the granulation tissue depth was analysed from the whole wound area. F) The schematic indicating 9 selected locations for measuring the granulation tissue depth as well as definitions of the outer (1 & 6), intermediate (2 & 5), central (3 & 4) and whole (1–6) wound regions. All data are presented as mean ± SEM. Statistical analysis is performed with Kruskall Wallace multivariate analysis followed by ad hoc two sample Mann Whitney U-test analysis). ns = non-significant, *p < 0.05, **p < 0.01, ***p < 0.001 and ****p < 0.0001.

**Fig 6:**
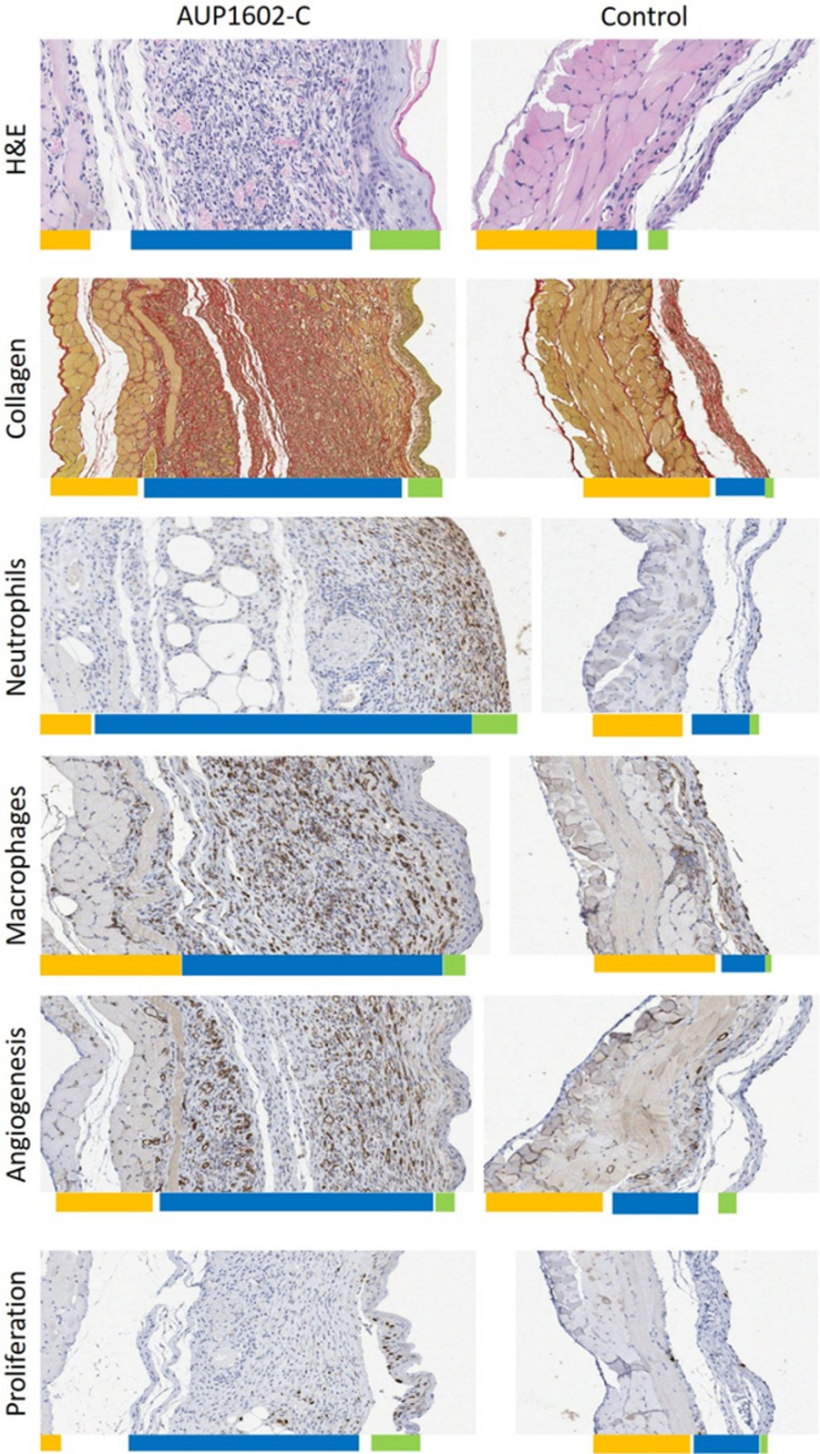
Example microscopic images of each histological and immunohistochemical staining. Wounds treated with AUP1602-C (left) or vehicle (right) with coloured bars below the sections approximating the thickness of the different tissue layers (yellow: muscle tissue, blue: granulation tissue and green: epithelium. A) H&E staining for GTP depth, B) PSR-staining for collagen deposition, C) anti-CD31-staining for angiogenesis, D) anti-F4/80-staining for macrophages, E) anti-NIMP-R14-staining for neutrophils and F) BrdU staining for cellular proliferation.

GTD was measured from nine longitudinal locations across the wound region. The analysed H&E-stained sections revealed that in the AUP1602-C treated wounds there was a significantly increased accumulation of granulation tissue in comparison to the vehicle group. The mean GTD in the AUP1602-C treated wounds was more than 200 μm thick in comparison to 50 μm in the vehicle group (Figs 5E and 6). The prevalence of other targets was assessed separately for the central, intermediate and outer regions of the wound as well as the cumulative total wound area (see Fig 5F for details). With the exception of macrophage prevalence in central, outer and cumulative whole wound regions, all other measured parameters were significantly increased in all wounds and regions after treatment with AUP1602-C in comparison to vehicle (Figs 5A–5D and 6). However, even the macrophage marker was present in higher amounts in wounds treated with AUP1602-C than with vehicle, despite not being statistically significant. Altogether, this indicates an active recruitment of inflammatory cells to the wound site and confirms the initiation of angiogenesis and fibroblast proliferation within the wound region at a microscopic scale. In general, the treatment effect was stronger when measured in the central region of the wound, whereas closer to the edges of the wounds the differences between AUP1602-C and vehicle were weaker.

### Dose finding study

After showing initial efficacy of AUP1602-C LBP treatment and in preparation for the safety and toxicology study, we investigated different dose concentrations and administration frequencies in order to define the most optimal dosing regimen. In this dose finding study the same diabetic db/db mouse model as described above for delayed wound healing was used and AUP1602-C was applied at four dose levels: 5x10^6^, 5x10^7^, 5x10^8^ and 5x10^9^ CFU/mL. Furthermore, three dosing frequencies were used to keep the cumulative total bacterial count per treatment period similar in all groups (1.5–1.75x10^9^ total CFU): daily for 7 days, every other day until day 12, and every fourth day until day 20. The data gathered from the different AUP1602-C treatment regimen were compared to data from wounds on control animals, which had been treated with the vehicle alone daily for 1 week.

**Fig 7:**
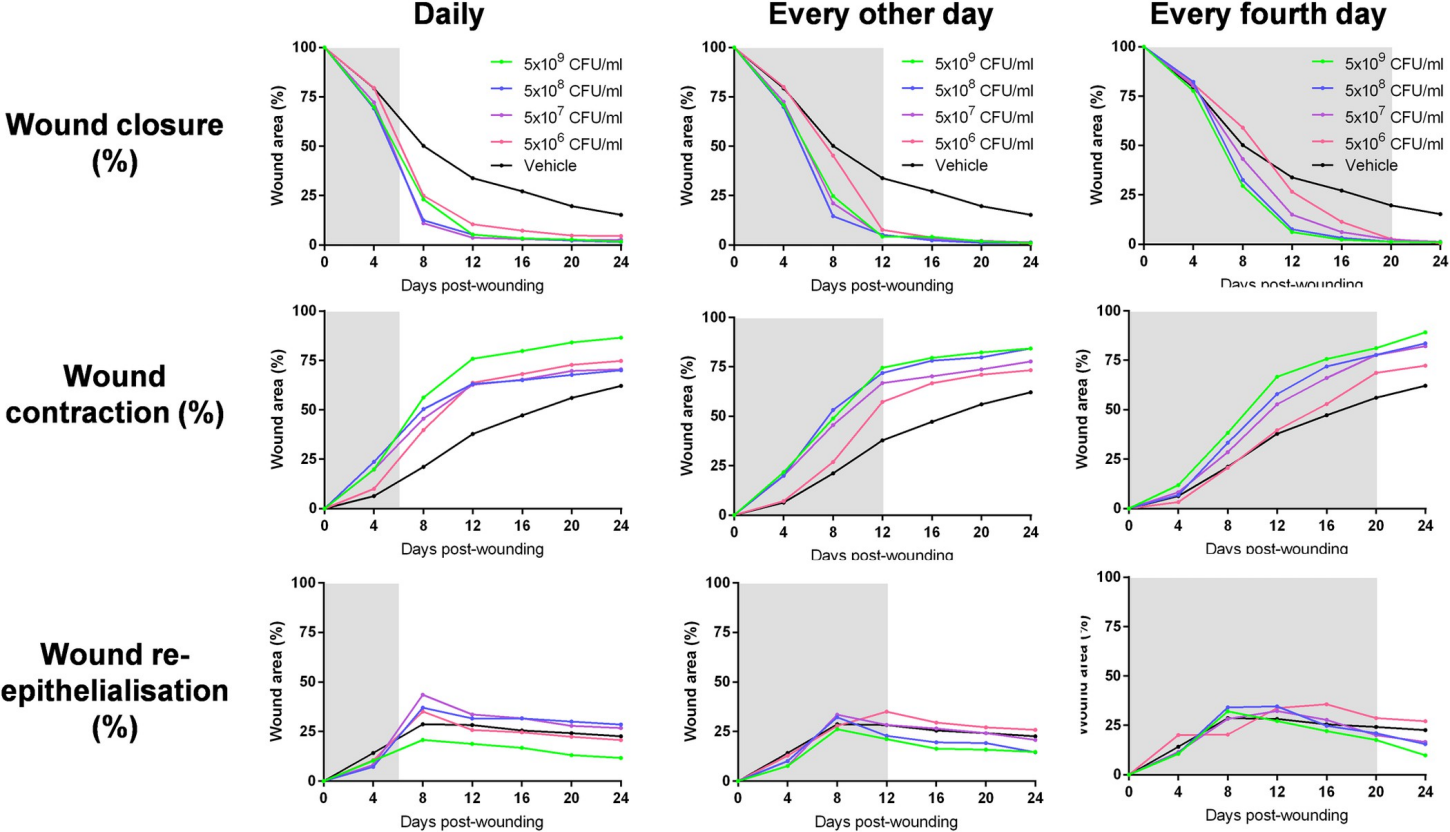
Measurements related to *in vivo* wound healing efficacy with variety of dosing regimen. Wound closure (top row), contraction (middle row) and re-epithelialisation (bottom row) in db/db mice after administrating doses between 5x10^6^ and 5x10^9^ CFU/ml of AUP1602-C either on daily basis until day 6 (left column), every other day until day 12 (middle column) or every fourth day until day 20 (right column). Grey background indicates treatment period. Data is presented as group mean.

**Fig 8:**
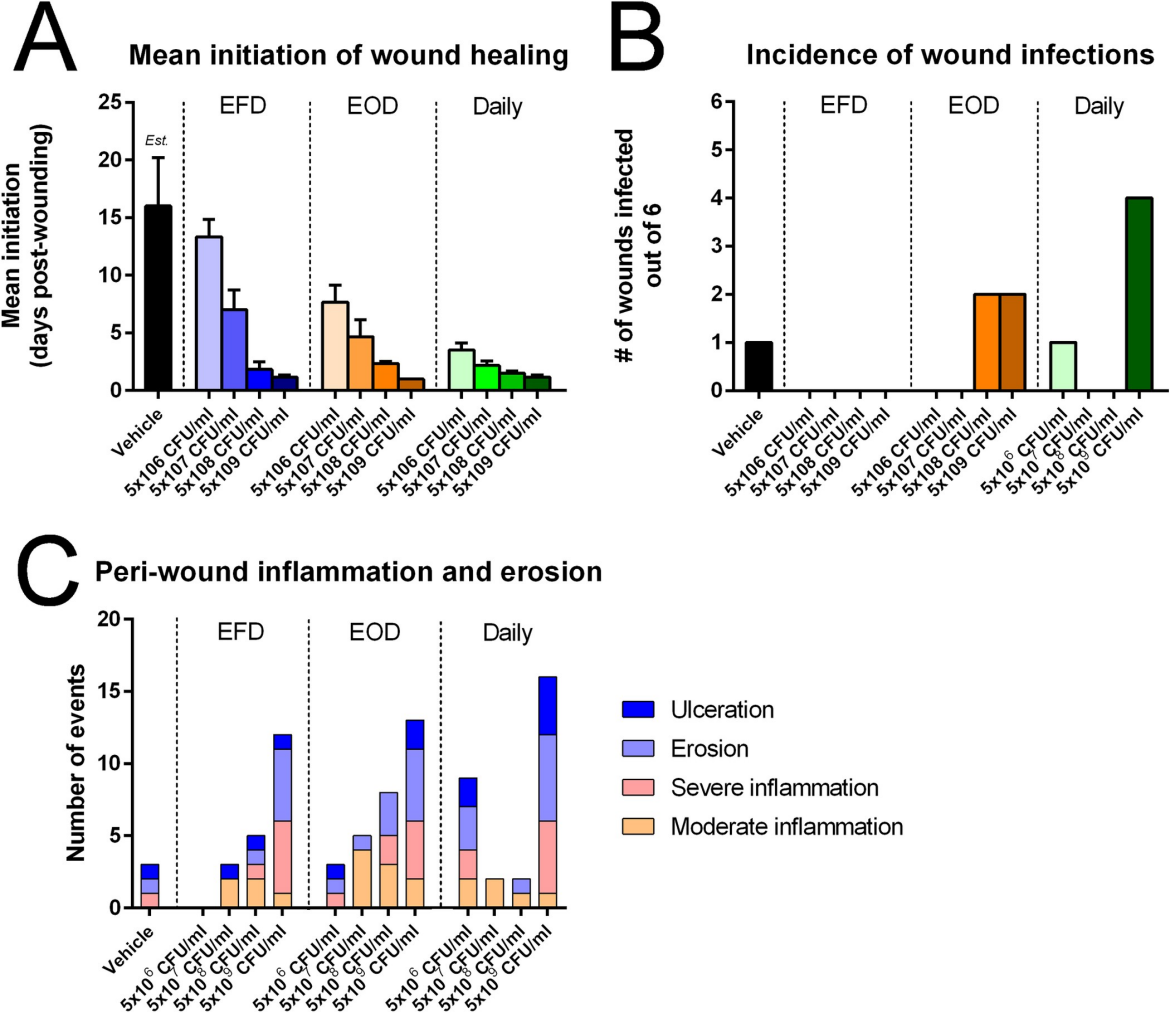
Results and observation of the dose finding study. A) The mean initiation of the wound healing after each dose and frequency combination, B) incidence of wound infections during the study and C) the peri-wound inflammation and erosion events observed in the animals. Data presented as mean ± SEM (A) or as individual number of findings (B & C). The data were not statistically analysed. Est. = estimated mean value for vehicle wound healing initiation as most of the wounds were not healing by the end of the study. For such wounds, the termination date (day 20) was used as value for the calculations.

Comparable to the initial *in vivo* efficacy study, all of the AUP1602-C treatment regimens in the dose finding study were found to accelerate wound healing in the db/db diabetic mouse model (Fig 7). Treatment of wounds with AUP1602-C led to significant increases in overall wound closure and wound contraction, including significant improvements in the angiogenic response (visual assessment) and in the initiation of neo-dermal tissue formation relative to wounds that had been treated with the vehicle alone. The rate of wound healing initiation was positively associated with the exposure to AUP1602-C. Greater exposure to AUP1602-C in bacterial cell concentration and frequency of treatment resulted in a more rapid initiation of neo-dermal tissue formation (Fig 8A).

Furthermore, during the study the wounds were also examined for clinical signs of infection and peri-wound inflammation. Several wounds were considered to have a contamination/infection but in general, the rate of wound infections was low. The root cause of the infections was not followed up during the study as *Lactococcus lactis* is not considered as pathogenic species thus most likely not the main cause of the infections. A higher incidence of the potential infections was observed in two of the three regimens of the highest dose concentration group (5x10^9^ CFU/ml) (Fig 8B) and stronger peri-wound inflammation was generally correlated with higher dose concentrations of AUP1602-C (Fig 8C). One possible explanation for these observations could be that both infection and inflammation were related to excessive levels of un-managed wound exudate present on the wounds and the peri-wound skin.

The purpose of the dose finding study was to determine an optimum dose (concentration) and dose frequency for the application of the AUP1602-C LBP. Through a systematic selection process based on the available data about wound healing efficacy parameters, the number of suspected wound infections and peri-wound inflammation an optimal dosing regimen was chosen. The following summarizes the selection process: the highest dose concentrations (5x10^9^ CFU/ml–all frequencies) were rejected because of their propensity to cause peri-wound inflammation. EFD dosing regimens were rejected because they were less effective for promoting wound closure in comparison to higher dosing frequencies. The dose concentration of 5x10^6^ CFU/ml daily and EOD were rejected due to poor wound closure relative to other doses/frequencies. The remaining four regimens (5x10^7^ and 5x10^8^ CFU/ml administered either daily or EOD) were largely similar in terms of their impact on wound healing and thus the combination of the least exposure to the AUP1602-C LBP in the longest timespan (i.e. 5x10^7^ CFU/ml EOD in order to minimize exposure to LBP at any given moment during the treatment) was considered the minimal effective dose to promote wound healing in the db/db mouse delayed healing model.

### GLP safety and toxicity study

Using the minimal effective dosing regimen the GLP safety and toxicity study for the treatment for full thickness wounds with the AUP1602-C LBP was performed in the Göttingen SPF minipig full thickness wound model. The wounds were treated either with AUP1602-C, vehicle or commercial 0.9% sterile saline. The treatment was performed either using the optimal dose regimen of 5x10^7^ CFU/ml EOD or with the higher dose of 5x10^9^ CFU/ml EOD in order to maximize the exposure. All wounds were treated with a total of 7 applications of the respective treatment. One half of the animals were sacrificed after finishing the treatment regimen whereas the other half were kept for another 14 days as a recovery group.

**Fig 9:**
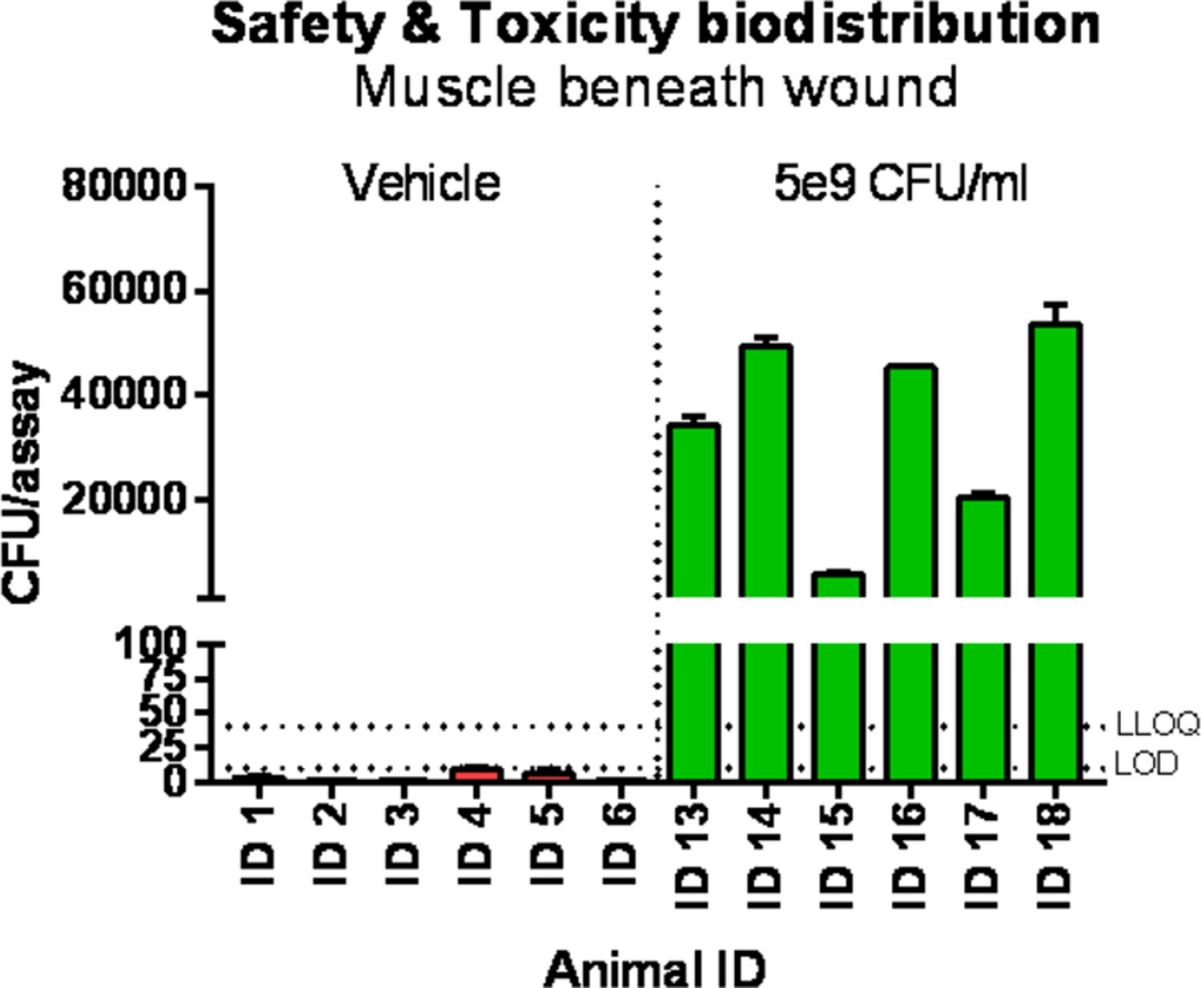
Biodistribution of AUP1602-C determined by qPCR in wound bed. Samples collected at day 14 indicating clear exposure to the bacterial construct. Data presented as mean ± SEM. Data not statistically analysed. LLOQ = lower limit of quantitation (40 CFUs), LOD = lower limit of detection (20 CFUs).

The safety and toxicity study did not show any serious adverse events nor any effects on body weight or food consumption during the study. Furthermore, no treatment related adverse events were observed in the ophthalmoscopic examination, in the clinical pathology parameters (haematology, clinical chemistry and urine), in ECG recordings, in organ weights, in macroscopic observations at necropsy and in the histopathology (Table 2). All wounds healed and contracted as expected and only minor differences were observed in the macroscopically assessed wound healing parameters. The observed differences reflect the normal wound healing variation observed in healthy minipigs. Furthermore, the SPF Göttingen minipig full thickness wound model is not a model for a delayed wound healing and all wounds were expected to heal regardless of the treatment. The histopathological evaluation did not reveal any treatment related negative systemic changes and the process of healing was similar in all wounds. Investigation of biodistribution and shedding of AUP1602-C by qPCR did not show any significant patterns of AUP1602-C distribution to other organs (blood, skin, liver, mandibular, mesenteric, cervicalis, inguinalis and axillaris lymphanoids, parotid, lacrimal, sublingual and -mandibular glands and heart valves) or shedding in urine and faces. However, the AUP1602-C exposure was clearly observed in the muscle tissue below the wound (Fig 9) with a noticeable difference in accumulation pattern between the 5x10^7^ and 5x10^9^ CFU/ml doses.

**Table 2:** Summary of the 14-day repeated-dose toxicity study in minipigs with full-thickness wounds with a 14-day recovery period.

**Species/strain**	Göttingen minipigs					
**Initial age**	9–12 months					
**Dose levels**	0 CFU/ml, 5x10^7^ CFU/ml, 5x10^9^ CFU/ml					
**Duration of dosing**	14 days, treatment on every other day					
**Duration of Postdose**	14 days					
**Method of administration**	Topical on wounds					
**Vehicle/Formulation**	5% dextrose, 2.5 sodium chloride, 1.6% sodium acetate					
**GLP Compliance**	Yes					
**Parameters controlled**	Body weight, food consumption, body temperature, clinical observations, opthalmoscopy, electrocardiography, haematology, clinical chemistry, urinalysis, organ weight, gross pathology, histopathology					
**Special feature**	Biodistribution and macroscopic wound evaluation					
**Brief conclusion**	Topical administration with 5x10^7^ and 5x10^9^ CFU/ml of AUP1602-C in surgically established full thickness wounds at 7 occasions during a time span of 14 days was well tolerated and did not cause any treatment releated systemic changes in male and female Göttingen minipigs. All wounds healed as normal and the wound healing process was advanced in all wounds at the end of the main study period and the recovery period.					
**NOAEL**	1x10^9^ CFU/ml					
**Dose (CFU/ml)**	0 (Control)	5x10^7^	5x10^9^	0 (Control)	5x10^7^	5x10^9^
**Number of animals**	M: 5	M: 5	M: 5	F: 5	F: 5	F: 5
**Number of wounds**	4	4	4	4	4	4
**Noteworthy findings**	No noteworthy findings wound in the study					

## Conclusions

The recombinant LBP AUP1602-C has been designed as a single therapeutic entity based on the non-pathogenic lactic acid bacterium *L*. *lactis* subspec. *cremoris* that has been genetically engineered to produce the human cytokines and growth factors FGF-2, IL-4 and CSF-1. AUP1602-C addresses different aspects of wound healing simultaneously, namely fibroblast proliferation, angiogenesis and immune cell activation, which are distorted in the chronic inflammatory wounds. The individual treatment factors combined in AUP1602-C and produced in the wound micro-environment aim at initiating the physiological wound healing process in chronic wounds such as DFU. Effectively, the FGF-2 is a pleiotropic growth factor involved in controlling development and physiological processes that play a major role in the activation of wound healing. By promoting endothelial cell-, fibroblast- and keratinocyte proliferation and migration FGF-2 stimulates angiogenesis, granulation, and epithelization [45, 46].

IL-4 is an immunoregulatory and anti-inflammatory cytokine that directs macrophages towards the wound-healing alternate M2 phenotype [47, 48]. Apart from its effects on immune cells, IL-4 also enhances the synthesis of extracellular matrix proteins such as collagen and fibronectin by fibroblasts [49] and promotes their migration [50], it induces the proliferation of keratinocytes [51], the differentiation of myofibroblasts [52] and decreases biosynthesis of matrix metalloproteinase [53]. Evidence suggests that IL-4 may also promote angiogenesis through its actions on endothelial cells [54].

CSF-1 is an immunoregulatory protein that is involved in the recruitment of myeloid derived cells from the circulation and their differentiation and it is also essential for the migration, proliferation, activation, survival and polarization of monocytes/macrophages into M2 phenotype [55–58]. Furthermore, there is evidence to suggest anti-inflammatory and tissue repair functions of CSF-1 [57], and indirect promotion of angiogenesis [59]. Finally, also the lactococcal host cells themselves are known for their immuno-stimulating and adjuvant properties [60, 61].

In the *in vivo* studies presented in this publication, the recombinant multi-factor LBP AUP1602-C was found to promote wound repair with a range of dose concentrations and frequencies in the db/db diabetic mouse model–a widely accepted animal model of delayed wound healing. Treatment of wounds with AUP1602-C led to a significant increase in overall rate of wound closure. Detailed analysis showed that all aspects of wound healing have been affected by the treatment such as the speed of the initiation of wound healing, wound contraction, wound re-epithelialisation, angiogenic response, collagen deposition, recruitment of phagocytic cells and cellular proliferation. Interestingly, the wounds on non-diabetic mice close predominantly by contraction while those on db/db diabetic mice have a significantly reduced ability to contract probably due to impoverished granulation tissue formation [62]. As a result, wounds on diabetic animals tend to close to a greater extent by re-epithelialisation than those on non-diabetic animals. The forces, that drive the process of contraction, are thought to derive from the activities of fibroblasts that populate the neo-dermal compartment of cutaneous wounds. The observation of enhanced wound contraction after AUP1602-C treatment suggests improvement in granulation tissue function which may in turn be explained by an increase in the speed and amount of formation of granulation tissue, or increased contractile capacity of the tissue.

The significant improvements in the initiation of wound healing based on visual assessment of the angiogenic response in the wound clearly correlated with the AUP1602-C dose: as more AUP1602-C was administered either by concentration or by dosing frequency, the wounds shifted into a healing state more rapidly. Some limitations of the treatment regimens were noted as high exposure to AUP1602-C either by frequency or dose also tended to promote elevated peri-wound inflammation. This appeared to have a negative impact on wound closure, although also in this situation AUP1602-C treatment remained superior to the vehicle.

The GLP safety and toxicity study further indicated that the AUP1602-C regimen was well tolerated and that there were no indications of treatment related systemic toxicity in any study group or in any safety related end points, including clinical signs, body weight, food consumption, ECG, ophthalmoscopy, haematology, clinical chemistry, urinalysis, organ weights, macroscopic observations at necropsy and histopathology observations. The good safety profile can be ascribed to the local administration within the wound region, which limited biodistribution and therefore lack of systemic exposure of the AUP1602-C LBP. Since there was no relevant biodistribution of AUP1602-C from the site of application to other body fluids or tissues, there was also no detectable shedding in urine or feces. Similar to observations in the mice efficacy studies, some inflammation of the wound edges was observed in all study groups. The severity of the inflammation was slightly more pronounced in wounds treated with AUP1602-C than in vehicle or saline treated, however, the healing process was considered within the normal range of wound healing both macroscopically and histopathologically.

In summary, we demonstrate *in vivo* using a recognized animal model for delayed wound healing, that the synergistic effect caused by *in situ* produced proteins together with the intrinsic activity of the AUP1602-C LBP leads to faster wound closure, faster wound contraction, increased neo-dermal tissue generation in wounds, increased angiogenic response in wounds, and thus accelerated wound healing. Furthermore, the nonclinical safety studies demonstrate that AUP1602-C is well tolerated at all tested doses and schedules and no major toxicity or dose-related systemic effects are observed even though mild local site reactions (peri-wound erythema, edema, peri-wound inflammation) can be somewhat expected but may also point towards an ongoing wound healing process. Therefore, DFU patients suffering from an impaired wound healing process or chronic wounds could benefit from treatment with AUP1602-C leading to re-initiation and/or faster and complete wound healing. Faster wound healing and closure would also lead to a lower risk for infection and other complications (e.g. odor) as well as less pain, which ultimately would significantly increase the quality of life of those patients.

Currently, the standard therapeutic management of diabetic wounds is focused on local wound care by assurance of adequate perfusion, debridement to remove non-viable tissue, provision of proper off-loading, control of infection and local wound care by applying a dressing that matches the current state of the wound (e.g. exudate control, moisturizing) performed by a multidisciplinary team. Advanced therapies for chronic wounds include devices or products that could speed up healing compared to the standard wound care, such as hyperbaric oxygen therapy, electrical stimulation, negative wound pressure therapy, special purpose dressings, skin grafts, bioengineered skin, drugs or biologics, as e.g. locally administered growth factors. Despite these strategies, the rate of amputation in diabetic patients with wounds such as chronic foot ulcers remains very high [63, 64]. Moreover, no pharmaceutical product has gained marketing authorization approval for DFU in more than 10 years. Thus, new strategies to promote wound healing in diabetics are urgently needed. The development and successful preclinical *in vivo* testing of the recombinant multifactor LBP AUP1602-C constitutes as such a new strategy. Based on the efficacy of AUP1602-C in the initiation of wound healing and complete wound closure and its good safety profile, the first-in-human multicentre clinical Phase I study is currently ongoing. The clinical Phase I study is conducted in clinical centres in Germany and Poland with the aim to evaluate safety, tolerability and efficacy of single and repeated doses of AUP1602-C as topical treatment in subjects with non-healing chronic DFU.

As a novel live biotherapeutic treatment strategy, there are both risks and advantages to AUP1602-C LBP based treatment of patients. In addition to known local site reactions, which are somewhat expected, there are also risks due to bacterial translocation or colonization potentially leading to AUP1602-C related local or general infections. Bacterial colonization and related biofilm formation in chronic wounds is one of the key factors causing delayed wound healing and impairment of the wound healing process into the inflammatory phase [3, 65, 66]. Up to 80% of chronic wounds contain bacterial material structures in a biofilm, as e.g. caused by *Staphylococcus aureus* and *Pseudomonas aeruginosa*, two of the most common wound pathogens. Besides direct tissues damage, the presence of pathogens in a wound also attract leukocytes further enabling the expression and/or secretion of multitude of inflammatory agents such as cytokines, proteases and reactive oxygen species. They all are contributing to the initiation and maintenance of inflammatory cascades, which affect not only the microbes in the wound but also the wound tissues and therefore are greatly impairing the wound healing [11, 67]. Although *L*. *lactis* subsp. *cremoris* is a non-pathogenic species [68], there have been reports of very rare cases of opportunistic infections mostly in patients with predisposing conditions, such as underlying disease, immunocompromised status or extremes of age [69, 70]. There have also been a few cases of patients with endocarditis related to systemic exposure to *L*. *lactis* [71–74]. Current *in vivo* data with AUP1602-C indicate that systemic biodistribution and shedding are minimal. However, in rare cases a bacterial translocation from the wound of a patient could occur into another organ. This could either worsen existing disease or even cause new symptoms (e.g. diabetic retinopathy or nephropathy should eyes or kidneys be targeted, respectively). Finally, being a live biopharmaceutical product, treatment with AUP1602-C as such does intrinsically cause inflammation when applied to the wound and a risk to increase and/or prolong the inflammatory stage thereby delaying the wound healing process does exist. Although the very same mechanism is also intended for AUP1602-C functionality (i.e attraction of suitable cell population for modulation of the wound microenviroment assisted by the expression/secretion of the cytokines) the initiation of the healing process should be monitored during the treatment.

In addition to the hazards related to bacterial growth, there are also potential risks in hypersensitivity/immunogenicity related adverse events that could occur due to AUP1602-C related antigens in patients after receiving the treatment. Both local and/or systemic reactions, including delayed reactions could occur in patients treated with AUP1602-C potentially causing loss of wound healing efficacy as the LBD would be rapidly neutralized or they could cause in the worst case even a life-threatening anaphylaxis. Current safety data from animal studies do not show immunogenicity related adverse events, also not after repeated topical dosing of AUP1602-C suggesting minimal systemic exposure limiting the probability and severity of immune-mediated adverse events. Development of treatment emergent anti-drug antibodies after topical administration of AUP1602-C could be possible, however, currently there is no presumption that these would be clinically relevant.

Despite the risks over the novel LBP therapy, in the benefit of AUP1602-C it has many advantages over other approaches such as classical small molecules, biologics. existing GMOs and gene therapy or their combinations. In comparison to many other available products and advanced wound healing products, AUP1602-C is the a true multi-factor therapeutic product consisting of a single bacterial bioreactor that induces an immune response itself and produces three wound healing cytokines and growth factors that are secreted directly into the wound micro-environment. This approach does not only avoid systemic administration and its accompanying side-effects but also addresses the complexity of factors that contribute to the initiation and full healing of a wound. This new strategy can also be used for other illnesses that require a multi-factor approach.

In addition to the good safety profile as shown in the safety and toxicity study, *L*. *lactis* is used in many products that have been classified by the US FDA as a Generally Regarded As Safe (GRAS) organism and it has from the EFSA the Qualified Presumption of Safety (QPS) status which was renewed in 2017 [75]. *L*. *lactis* is one of the best characterized Gram-positive bacteria with more than 100 years’ worth of physiological data, 50 years of genetics research and genetic engineering experience, 20 years of genome characterization and genomic and metabolic modelling [76]. It is also traditionally the main bacterial species used in starter cultures for the production of cheese and butter milk and has been used and domesticated by humans for thousands of years. *L*. *lactis* is consumed on a daily basis by millions of people and reports of proven or suspected infections by *L*. *lactis* strains are extremely rare and manageable by the use of antibiotics [69, 70] and thus AUP1602-C is superior to many alternative advanced wound care therapies, GMOs or gene therapy approaches when it comes of safety of the patient and considerations of GMO environmental control.

Lactococci have a long history of large-scale manufacturing as starter cultures for the dairy industry and thus there is much experience and knowhow concerning the production process and control as well as scale-up processes [77]. Furthermore, as the production of the therapeutic proteins happens *in situ* in the wound, it therefore makes costly downstream processing and protein purification completely unnecessary. This means that for a combination product only one process is needed instead of producing each therapeutic protein separately and then combining them into one product. Therefore, the production of the recombinant multi-factor LBPs is highly scalable and cost-efficient in comparison to the production of e.g. viral vectors, cell therapies and other advanced therapy medicinal products (ATMPs).

The bacterial platform in which the AUP1602-C is based, could also be used in the future for multiple other diseases and conditions as the platform itself is widely adjustable. For example, it is capable of producing large and heterogenic proteins that are notoriously difficult or even impossible with viral vectors. Secondly, the ability to produce and secrete multiple proteins simultaneously from single construct can make regulatory approaches concerning combination therapies much more feasible. Finally, as the product is a bioreactor capable of independently expressing and secreting the proteins, no systemic application of the product is required nor is there a need for specific target host cells, but in many of the cases a local administration to the pathogenic site is sufficient to ensure exposure to the multitherapy. This approach also makes the use of the biotherapeutic a simple non-surgical alternative to medical devices, special handling or specialised clinical teams.

## Supporting information

S1 Appendix Wound healing efficacy of AUP1601. During the development of AUP1602-C, a variant of the product named AUP1601 was studied for wound healing efficacy against control bacteria that do not express the target proteins and PBS sham treatments. Whilst the control bacteria alone had a significant effect on the would healing in the diabetic db/db mouse model for delayed wound healing, the experiment showed that the synergy of both the bacteria and the secretion of FGF-2, IL-4 and CSF-1 in the wound had better healing results. All data are presented as mean ± SEM. Statistical analysis is performed with Kruskall Wallace multivariate analysis followed by ad hoc two sample Mann Whitney U-test analysis). *p < 0.05, **p < 0.01, ***p < 0.001 and ****p < 0.0001 when compared against control bacteria group; ¤p < 0.05, ¤¤p < 0.01, ¤¤¤p < 0.001 and ¤¤¤¤p < 0.0001 when compared against PBS sham group.
(TIF)

S2 Appendix Raw data files. Excel containing all raw data from the studies presented in this publication.
(XLSX)

S3 Appendix Western blot original uncropped images. (TIF)
