# Supplementary figures and images for: Four in one—Combination therapy using live Lactococcus lactis expressing three therapeutic proteins for the treatment of chronic non-healing wounds
Source: PLoS One. 2022 Feb 28;17(2):e0264775. doi: 10.1371/journal.pone.0264775 (PMC8884502; doi:10.1371/journal.pone.0264775)

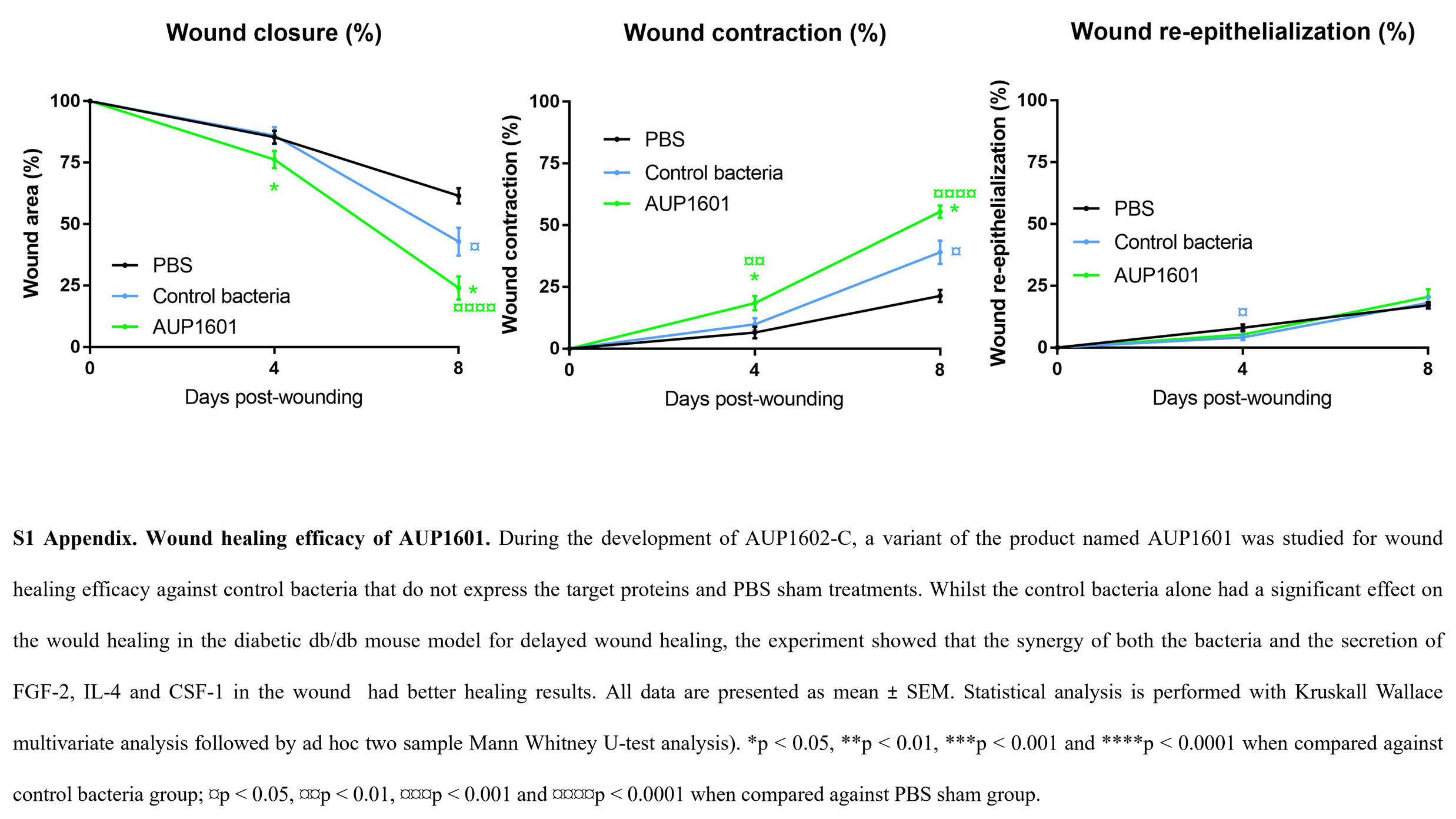

Supplement: S1 Appendix — During the development of AUP1602-C, a variant of the product named AUP1601 was studied for wound healing efficacy against control bacteria that do not express the target proteins and PBS sham treatments. Whilst the control bacteria alone had a significant effect on the would healing in the diabetic db/db mouse model for delayed wound healing, the experiment showed that the synergy of both the bacteria and the secretion of FGF-2, IL-4 and CSF-1 in the wound had better healing results. All data are presented as mean ± SEM. Statistical analysis is performed with Kruskall Wallace multivariate analysis followed by ad hoc two sample Mann Whitney U-test analysis). *p < 0.05, **p < 0.01, ***p < 0.001 and ****p < 0.0001 when compared against control bacteria group; ¤p < 0.05, ¤¤p < 0.01, ¤¤¤p < 0.001 and ¤¤¤¤p < 0.0001 when compared against PBS sham group. (TIF) [file pone.0264775.s001.tif]

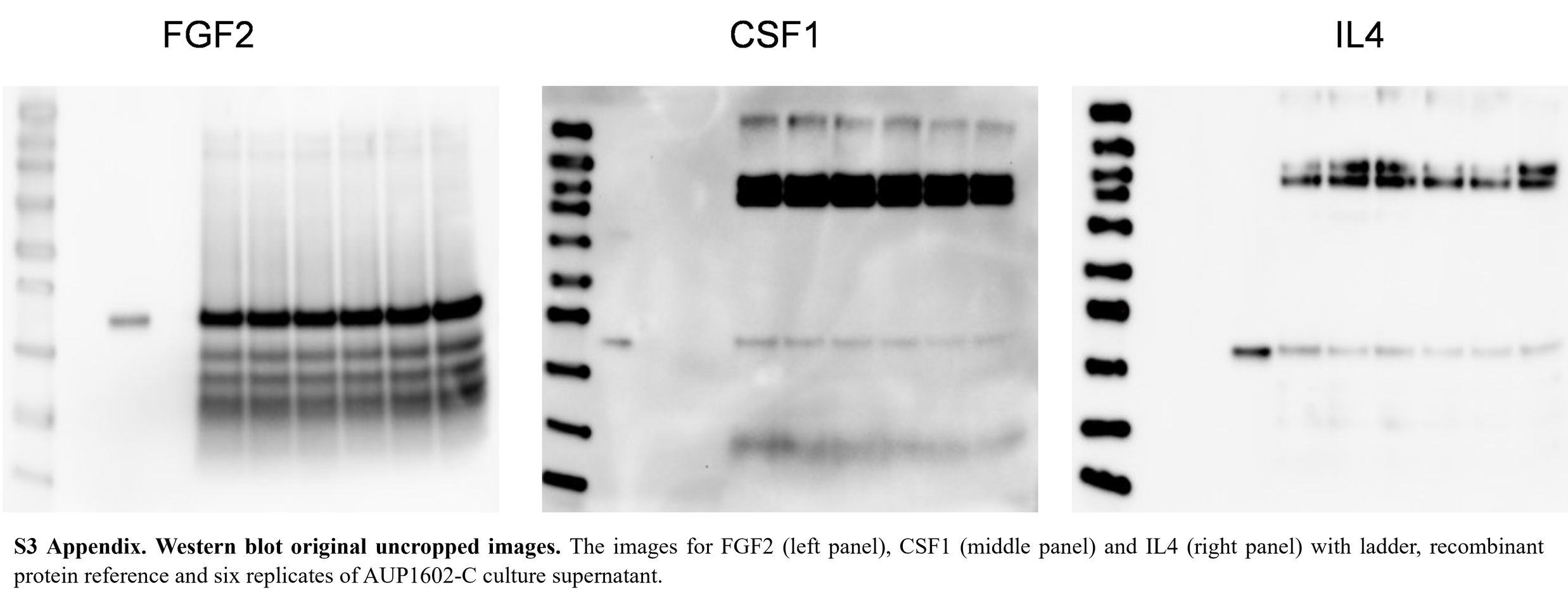

Supplement: S3 Appendix — (TIF) [file pone.0264775.s003.tif]
